# Serotonin sets up neutrophil extracellular traps to promote neuroendocrine prostate cancer metastasis in the liver

**DOI:** 10.1172/JCI191687

**Published:** 2025-04-15

**Authors:** Dean G. Tang

**Affiliations:** 1 Department of Pharmacology and Therapeutics, Roswell Park Comprehensive Cancer Center, Buffalo, New York, USA.; 2 Experimental Therapeutics Graduate Program, University at Buffalo and Roswell Park Comprehensive Cancer Center, Buffalo, New York, USA.

## Abstract

Castration-resistant prostate cancer frequently metastasizes to the liver, and prostate cancer liver metastases often present a neuroendocrine phenotype (i.e., neuroendocrine prostate cancer [NEPC]), but the underlying molecular underpinnings remain unclear. In this issue of the *JCI*, Liu et al. demonstrate that the neurotransmitter serotonin (also known as 5-hydroxytryptamine), produced by NEPC cells, gained access to and activated neutrophils by modifying histone 3 (H3) to form neutrophil extracellular traps, which in turn promoted NEPC macrometastases in the liver. The study suggests that blocking serotonin transport to neutrophils and inhibiting the enzymes that catalyze serotonin-mediated H3 modifications may represent alternative approaches to treating prostate cancer liver metastases.

## Increased prostate cancer liver metastasis in the clinic

**Figure 1:**
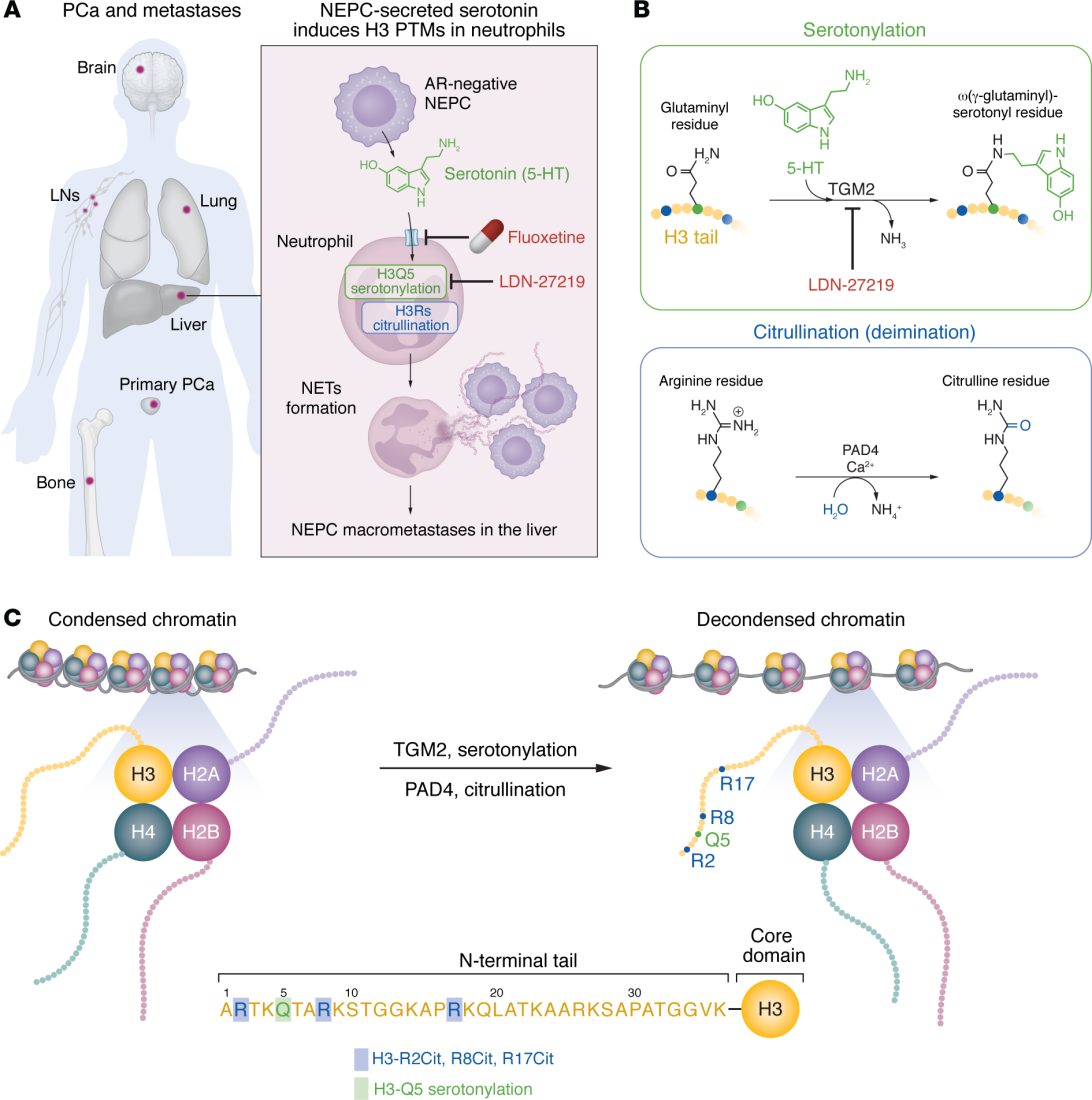
Serotonin promotes NEPC metastasis in the liver via posttranslational modification of H3 in neutrophils and increases NET formation. (**A**) Primary PCa can metastasize to different parts of the body including bone, LNs, brain, lungs, and liver. The findings from Liu et al. (7) support a model in which AR^–^ NEPC cells disseminate to the liver and secrete serotonin, which is taken up by neutrophils via SERT. Once in the neutrophils, serotonin mediates H3 serotonylation and citrullination, leading to neutrophil activation, NET formation, and subsequent NEPC macrometastasis in the liver. Notably, the antidepressant fluoxetine can inhibit SERT and may block liver metastasis. (**B**) The process of serotonylation is mediated by TGM2, while citrullination is mediated by PAD4. (**C**) Modification of core histones within the condensed nucleosome at H2A, H2B, H3, and H4 affects chromatin accessibility. Both posttranslational processes, serotonylation and citrullination, occur in the H3 N-terminal tail and act together to decondense and loosen chromatin. In particular, serotonylation modifies the fifth H3 residue Q5, and citrullination affects 3 arginine residues (i.e., R2, R8, and R17).

Prostate cancer (PCa) is a major malignancy claiming a high mortality rate worldwide. The majority of PCa is diagnosed as adenocarcinomas (i.e., prostate adenocarcinoma [PRAD]) expressing the androgen receptor (AR), with less than 1% as de novo neuroendocrine PCa (NEPC), which is AR^–^. While primary NEPC is treated with platinum, taxol, and etoposide-based chemotherapeutics, patients with advanced PRAD (i.e., high-grade tumors ineligible for prostatectomy) are treated with AR-targeting agents with or without concurrent radiotherapy. AR-targeting therapies, also called AR signaling inhibitors (ARSIs) or AR pathway inhibitors (ARPIs), encompass androgen deprivation therapy (ADT), which aims to block androgen production from the testicles (using luteinizing hormone–releasing hormone [LHRH] agonists or antagonists) and adrenal glands (with abiraterone acetate) and includes AR antagonists (e.g., enzalutamide, apalutamide, darolutamide). Most patients with PCa initially respond well to ARSIs but become refractory to these treatments within two to three years and develop castration-resistant prostate cancer (CRPC) (1). Patients with CRPC frequently harbor metastasis (i.e., mCRPC) in different organs including the bones, lymph nodes (LNs), brain, liver, and lungs (Figure 1A).

Since the introduction of the potent ARSIs abiraterone acetate (Zytiga; FDA approval in 2011) and enzalutamide (Xtandi; FDA approval in 2012) into the clinic, there have been alarming increases in metastasis to visceral organs such as the liver and in AR^–^ mCRPC, which often presents a neuroendocrine (NE) phenotype (2–5) and is referred to as CRPC-NE or treatment-induced NEPC (t-NEPC). The clinical increase in CRPC-NE has been linked to treatment-induced PCa cell lineage plasticity (1, 2, 5, 6); however, the mechanisms underlying the notable uptick in CRPC-NE in the liver remain murky. In this issue of the *JCI*, Liu et al. (7) provide interesting evidence that the neurotransmitter serotonin (also known as 5-hydroxytryptamine [5-HT]) could be secreted by NEPC cells to communicate with neutrophils. The generation of neutrophil extracellular traps (NETs) by neutrophils may attract and trap additional PCa cells to establish macrometastases in the liver (7) (Figure 1A). Mechanistically, serotonin in the neutrophils induces two posttranslational modifications (PTMs) on histone 3 (H3), i.e., serotonylation and citrullination (also known as deimination), to activate neutrophils, promote NET formation (i.e., NETosis), and drive liver-specific NEPC (7) (Figure 1A).

## PCa liver metastases have an NE phenotype

Liu and colleagues previously established a murine PCa liver metastasis model by tail-vein injection of tumor organoids derived from the *Rb1* and *Trp53* double-knockout (termed *PbCre*^+^ *Rb1^fl/fl^*; *Trp53^fl/fl^*) PCa (8). Using this model and in conjunction with in silico findings of abundant neutrophils in human PCa liver metastases, the authors first validated the accumulation of Cd11^+^Ly6G^+^ neutrophils in PCa liver metastases (7). Immunofluorescence costaining revealed that the liver metastases of both murine and human PCa showed NE features (i.e., neural cell adhesion molecule 1^+^ [NCAM1^+^]), enrichment of neutrophils (i.e., positive for myeloperoxidase [MPO] for mouse or CD66b for human), and, importantly, the formation of NETs (positive for H3 citrullination [H3cit]). Strikingly, intraperitoneally injected DNase I reduced MPO^+^H3cit^+^ NETs and liver metastases and extended the survival of tumor-bearing mice, implicating the NETs in causally driving PCa liver metastasis. These observations in PCa are consistent with emerging roles of NETs in helping establish the premetastatic niche and driving the metastasis of many cancers to the liver, lungs, and other organs (9–13).

## Serotonin and serotonin-synthesizing enzyme in PCa liver metastasis

What could be driving increased NETosis in NEPC liver metastases? Liu, Zhang, and co-authors hypothesized that NEPC-produced serotonin, a tryptophan-derived biogenic monoamine and well-known neurotransmitter, may be one of the drivers (7). Supporting their hypothesis, the authors found human CRPC-NE had higher mRNA levels of serotonin-synthesizing enzyme tryptophan hydroxylase 1 (TPH1) compared with PRAD. In vitro studies revealed that exogenous serotonin stimulated NET production in mouse bone marrow–derived and human peripheral blood–derived neutrophils, an effect that, in the murine system, could be recapitulated by treating neutrophils with conditioned medium (CM) from their wild-type NEPC organoid but not from those depleted of *Tph1*. Importantly, the *Rb1* *Trp53* double-knockout tumor organoids with *Tph1* knockdown produced fewer liver metastases, which could be partly rescued by the readministration of serotonin to mice. Using additional human NE cancer cell line models (i.e., small cell lung cancer and medullary thyroid cancer), Liu and colleagues nicely showed that knocking down *TPH1* in these cells also decreased NET formation and liver metastasis (7). These studies evince serotonin as a metastasis-promoting oncometabolite and the serotonin-synthesizing enzyme TPH1 as a driver of NE cancer metastasis to the liver.

## Serotonin modifies histones in neutrophils

Enterochromaffin cells of the gastrointestinal (GI) tract produce approximately 90% of the serotonin in the human body, with platelets contributing about 8% and the serotonergic neurons in the brain providing the remainder, approximately 2%. Serotonin controls GI, cardiovascular, brain, and other organ functions via binding to one of the approximately 15 membrane serotonin GPCRs called 5-HT receptors (5-HTRs). Serotonin may also regulate biological processes such as platelet aggregation through an HTR-independent process, via the transglutaminase-dependent covalent protein modification known as serotonylation (Figure 1B). A recent study reported that serotonylation on H3 at the fifth glutamine residue (termed H3Q5ser) stabilizes H3 methylation at the fourth lysine residue (known as H3K4me3) and helps keep the chromatin in the open conformation (14). Liu and co-authors observed that serotonin entered neutrophils through the serotonin transporter (SERT) and, once inside the nucleus, was covalently linked to H3Q5 via the action of transglutaminase 2 (TGM2). This process led to neutrophil chromatin decondensation and the formation of NETs. Consequently, both the SERT inhibitor fluoxetine and the TGM2 inhibitor LDN-27219 abrogated serotonin-induced H3Q5ser and NETosis. In contrast, SB224289, an inhibitor of HTR1B, whose mRNA was most highly expressed in human NEPC, did not manifest any apparent effects. Subsequent studies implicated TGM2 as a key regulator of serotonin-driven NET formation and NEPC liver metastasis. Thus, *Tgm2^–/–^* neutrophils showed reduced H3Q5ser and H3cit and lost responsiveness to serotonin-induced NETs. Furthermore, a TGM2 inhibitor reduced the *Rb1*;*Trp53* tumor organoid–derived liver NEPC burden, and *Tgm2*^–/–^ recipient mice exhibited substantially reduced liver NEPC compared with *Tgm2* wild-type mice (7).

## H3Q5ser and H3cit cooperate to loosen chromatin in neutrophils

One of the key findings of Liu et al. (7) pertained to the cross interactions and reciprocally reinforcing effects between the H3 serotonylation and H3 citrullination. Specifically, TGM2 catalyzed H3Q5ser, and the peptidylarginine deiminase 4 (PAD4) catalyzed H3cit on arginine (R) residues, including R2cit, R8cit, and R17cit (Figure 1C). Thus, murine bone marrow neutrophils lacking TGM2 (*Tgm2*^–/–^) showed reductions in both modifications: H3Q5ser and H3cit. Similarly, the TGM2 inhibitor simultaneously reduced H3Q5ser and H3cit in neutrophils from the livers of tumor-bearing mice in vivo. In human HL60 granulocytes, the H3 site–specific mutation replacing Q5 with an alanine, also diminished H3Q5ser and H3cit. Moreover, inhibition of SERT with fluoxetine, or TGM2 inhibition with LDN-27219, concordantly reduced serotonin-induced H3Q5ser and H3cit. shRNA-mediated knockdown of TGM2 or PAD4, as well as treatment with the PAD4 inhibitor Cl-amidine also blocked both H3 PTMs. Defined biochemistry studies subsequently demonstrated that TGM2 and PAD4 physically interacted with each other, coordinating H3Q5ser and H3cit to drive concordant chromatin co-occupancy and chromatin decondensation (7).

## New avenues for liver NEPC treatment and outstanding questions

The findings from Liu et al. (7) suggest that interfering with the serotonin pathway in general (15), and serotonin-initiated neurotransmitter/neutrophil signaling in particular, may represent an alternative therapeutic strategy to tackle liver metastasis of NE cancers. In support of these findings, Liu and authors showed that the SERT inhibitor fluoxetine, a clinically used antidepressant, inhibited the liver metastasis of both NEPC and medullary thyroid NE cancer and reduced H3Q5ser and H3cit modifications (7). Interestingly, an earlier study by another group, through unbiased high-throughput drug screening, also uncovered fluoxetine as an inhibitor of *Pten*^–/–^;*Rb1*^–/–^;*Trp53*^–/–^ NEPC by antagonizing tumor cell–intrinsic Akt activity (16). In addition to blocking neutrophil serotonin uptake by SERT inhibitors, other approaches to stopping the serotonin-related NET signaling may include inhibition of serotonin production (e.g., TPH1 inhibitors) and release, serotonin-mediated H3 modifications by inhibition of TGM2 (e.g., LDN 27219 and KCC009), and serotonin-driven NETosis (e.g., PAD4 inhibition using Cl-amidine). In fact, TPH1 inhibitors, such as LP-533401, telotristat ethyl (LX1032), and p-chlorophenylalanine (pCPA), which primarily inhibit peripheral serotonin production in the gut and other peripheral tissues, are being investigated for treating carcinoid syndrome, inflammatory bowel disease, and NE tumors. And octreotide, an inhibitor of serotonin release, is being explored for the treatment of well-differentiated NE cancers (15).

The work by Liu et al. (7) solidifies the roles of neurotransmitters such as serotonin, adrenaline, and dopamine in the expanding list of oncometabolites (e.g., hydroxyglutarate, lactate, fumarate, succinate), which aid cancer progression by covalently modifying histones and other substrates, but also raises many interesting questions. For example, do patients with mCRPC involving the liver and other visceral organs have higher levels of serotonin in the circulation? Does the liver preferentially express the proteins (e.g., TPH1, SERT, TGM2) that regulate serotonin biogenesis and metabolism? Serotonylation can occur on many different protein substrates, so what dictates the specificity of H3Q5ser in neutrophils? How exactly do the NETs driven by locally NEPC-produced serotonin attract additional PCa cells? As CRPC-NE lesions are often of mixed phenotypes (4), do the NETs trap both AR^+^ adenocarcinoma and AR^–^ NEPC cells? With increasing appreciation of the critical roles that the nervous system plays in regulating PCa progression and NEPC development (17–19), could PCa-innervating nerves produce serotonin and other neurotransmitters, which then drive tumor cell–intrinsic signaling via neurotransmitter receptors, to facilitate the liver metastasis? Answers to these questions will open exciting avenues of research and lead to repurposed and combinatorial therapies for aggressive NE cancers and their liver metastases.

## Version 1 04/15/2025

Electronic publication

## References

[1] Tang DG (2022). Understanding and targeting prostate cancer cell heterogeneity and plasticity. Semin Cancer Biol.

[2] Liu C (2024). PROX1 drives neuroendocrine plasticity and liver metastases in prostate cancer. Cancer Lett.

[3] Bluemn EG (2017). Androgen receptor pathway-independent prostate cancer is sustained through FGF signaling. Cancer Cell.

[4] Labrecque MP (2019). Molecular profiling stratifies diverse phenotypes of treatment-refractory metastatic castration-resistant prostate cancer. J Clin Invest.

[5] Westbrook TC (2022). Transcriptional profiling of matched patient biopsies clarifies molecular determinants of enzalutamide-induced lineage plasticity. Nat Commun.

[6] Jamroze A (2024). Treatment-induced stemness and lineage plasticity in driving prostate cancer therapy resistance. Cancer Heterog Plast.

[7] Liu K (2025). 5-HT orchestrates histone serotonylation and citrullination to drive neutrophil extracellular traps and liver metastasis. J Clin Invest.

[8] Liu K (2021). 5-HT orchestrates histone serotonylation and citrullination to drive neutrophil extracellular traps and liver metastasis. Cell Prolif.

[9] Bojmar L (2024). Multi-parametric atlas of the pre-metastatic liver for prediction of metastatic outcome in early-stage pancreatic cancer. Nat Med.

[10] He XY (2024). Chronic stress increases metastasis via neutrophil-mediated changes to the microenvironment. Cancer Cell.

[11] Mousset A (2023). Neutrophil extracellular traps formed during chemotherapy confer treatment resistance via TGF-beta activation. Cancer Cell.

[12] Lee W (2025). Neutrophil extracellular traps promote pre-metastatic niche formation in the omentum by expanding innate-like B cells that express IL-10. Cancer Cell.

[13] Wang X (2024). NQO1 triggers neutrophil recruitment and NET formation to drive lung metastasis of invasive breast cancer. Cancer Res.

[14] Zhao S (2021). Histone H3Q5 serotonylation stabilizes H3K4 methylation and potentiates its readout. Proc Natl Acad Sci U S A.

[15] Balakrishna P (2021). Serotonin pathway in cancer. Int J Mol Sci.

[16] Chen L (2023). High-throughput drug screening identifies fluoxetine as a potential therapeutic agent for neuroendocrine prostate cancer. Front Oncol.

[17] Magnon C (2013). Autonomic nerve development contributes to prostate cancer progression. Science.

[18] Zahalka AH (2017). Adrenergic nerves activate an angio-metabolic switch in prostate cancer. Science.

[19] Braadland PR (2019). The β2-adrenergic receptor is a molecular switch for neuroendocrine transdifferentiation of prostate cancer cells. Mol Cancer Res.

